# Atypical Omenn Syndrome due to Adenosine Deaminase Deficiency

**DOI:** 10.1155/2012/919241

**Published:** 2012-05-31

**Authors:** Avni Y. Joshi, Erin K. Ham, Neel B. Shah, Xiangyang Dong, Shakila P. Khan, Roshini S. Abraham

**Affiliations:** ^1^Division of Pediatric and Adult Allergy and Immunology, Department of Pediatric and Adolescent Medicine, Mayo Clinic, Rochester, MN 55905, USA; ^2^Department of Internal Medicine, Mayo Clinic, Rochester, MN 55905, USA; ^3^Department of Medical Genetics, Mayo Clinic, Rochester, MN 55905, USA; ^4^Department of Laboratory Medicine and Pathology, Mayo Clinic, Rochester, MN 55905, USA; ^5^Division of Pediatric Hematology and Oncology, Department of Pediatric and Adolescent Medicine, Mayo Clinic, Rochester, MN 55905, USA

## Abstract

We present here a novel case of an atypical Omenn syndrome (OS) phenotype due to mutations in the *ADA* gene encoding adenosine deaminase. This case is noteworthy for a significant increase in circulating CD56^bright^CD16- cytokine-producing NK cells after treatment with steroids for skin rash.

## 1. Introduction

Omenn syndrome (OS) is a distinct manifestation of Severe Combined Immunodeficiency (SCID) characterized by erythroderma, hepatosplenomegaly, lymphadenopathy, eosinophilia, and elevated IgE and alopecia [1–3]. Patients with OS usually present in infancy with viral or fungal pneumonitis, chronic diarrhea, and failure to thrive, but in contrast to classic SCID patients also have the above-mentioned characteristics.

Here we present a case of atypical OS in an infant with adenosine deaminase (ADA) deficiency.

## 2. Case Report

**Figure 1:**
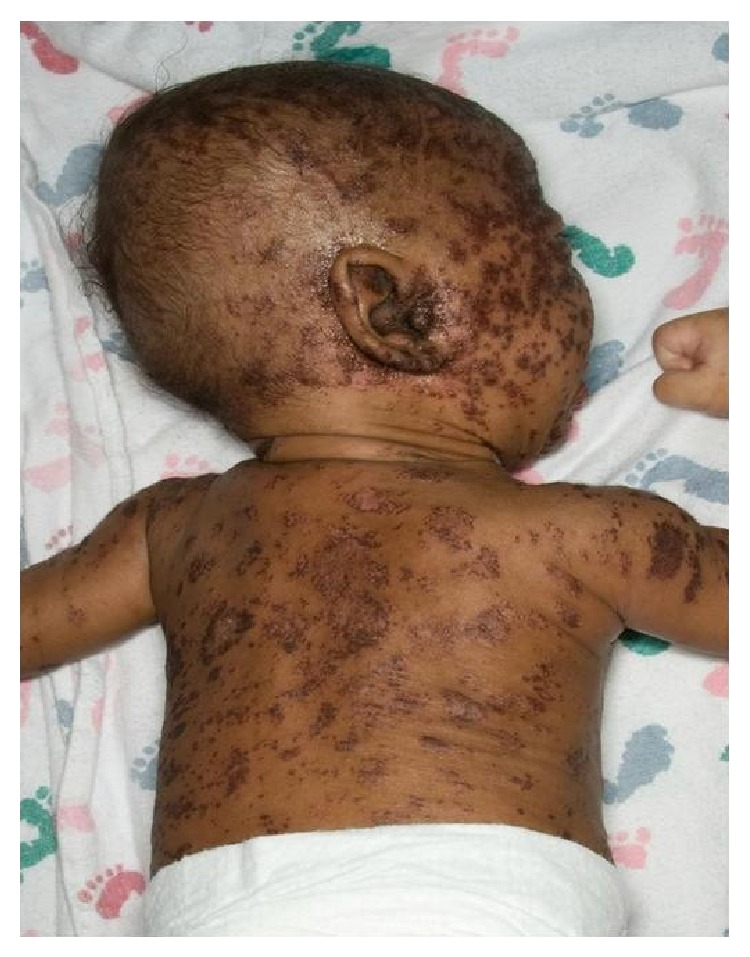
Diffuse rash in an infant with ADA deficiency.

A 2-month-old Somali boy, born to consanguineous parents, presented to our hospital with concerns for immunodeficiency. The initial symptoms included cough, diffuse skin rash (Figure 1), hepatosplenomegaly, and a sepsis-like syndrome. A complete sepsis workup was negative, except for an absolute lymphocyte count (ALC) of 0, which triggered evaluation for an underlying primary immunodeficiency. T-, B- and NK-cell quantitation in blood revealed a T-B-NK- phenotype consistent with Severe Combined Immunodeficiency (SCID). The molecular defect was likely thought to be in the *ADA* gene due to the ethnicity of the patient and the lymphocyte phenotype, therefore, ADA levels were tested and found to be 0 (reference range: 0.3–1.5/g Hb).

Broad-spectrum antibiotics were initiated, but with negative cultures, were maintained on prophylactic antibiotics (Bactrim for Pneumocystis, initial Fluconazole followed by Caspofungin for antifungal, IVIG and Palivizumab for RSV).

**Figure 2:**
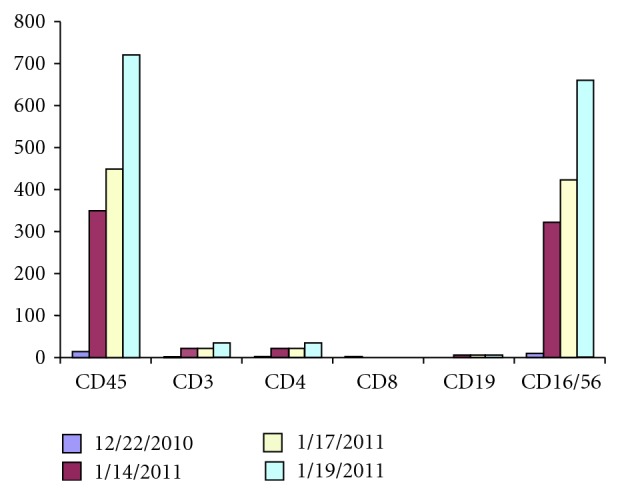
Serial T, B, and NK lymphocyte measurements.

His skin rash was extensively evaluated and the pathology was initially regarded as possible graft versus host disease due to maternal engraftment [4], due to the presence of apoptotic keratinocytes. However, the absence of circulating T cells argued against that possibility and chimerism [5] studies on a skin biopsy revealed all cells to be XY in origin, confirming absence of maternal engraftment. Treatment was initiated with steroids and PEG-ADA (biweekly, 60 U/kg/week due to the absence of a matched donor for stem cell transplantation and unsuitability at the time for gene therapy [6]; trough plasma ADA levels were monitored while on PEG-ADA treatment-range of 30–40 mmol/hr/mL (target levels > 12 mmol/hr/mL), but shortly thereafter, there was a remarkable increase in circulating lymphocyte counts with doubling in a span of 5 days. Lymphocyte immunophenotyping revealed these to be primarily NK cells (CD16/56), with a small number of T cells (Figure 2, PEG ADA started on 01/03/2011).

**Figure 3:**
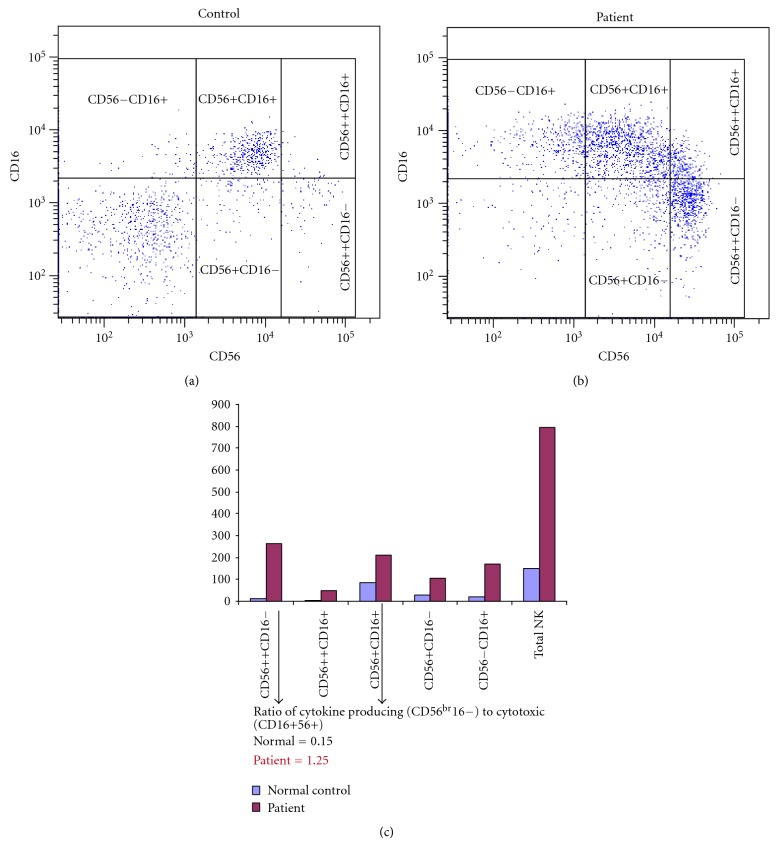
Natural Killer (NK) cell phenotyping.

Analysis of thymic function (CD4 recent thymic emigrants: CD4+ CD45RA+ CD31+) revealed an almost complete lack of naive T cells, contrary to what would typically be expected for age, and T cells that were present had the memory, CD45RO+ phenotype, as often seen in OS. Flow cytometric analysis of the NK-cell population revealed an unusual expansion of cytokine-producing NK cells, CD56^bright^CD16−, which is typically only 10% of circulating NK cells (Figure 3). The appearance of this unusually expanded NK cell subset and small numbers of CD45RO+CD4+ T cells in blood was likely related to retrafficking of these cells from the skin lesions rather than *de novo* generation due to ADA treatment due to the short time-interval posttreatment in which the cells were detected. There has previously been one report of skin-infiltrating CD56^bright^CD16− NK cells in a patient with X-linked (*IL2RG* mutation) SCID who had OS-like features [7].

The patient reported herein had several characteristics of OS as mentioned but was atypical for the presence of the expanded CD56^br^CD16− NK cells, normal IgE levels, and lack of eosinophilia.

Molecular analysis of the *ADA* gene in both parents revealed a heterozygous Q3X nonsense mutation (c. 7 C>T; CAG>TAG), which has been previously reported to be present at a frequency of at least 1 in 5000 to 1 in 10,000 in the Somali population [8].

 Despite use of high-dose steroids, his respiratory status worsened and he developed seizures. A spinal tap showed evidence of human herpes virus-6 (HHV 6) via PCR with HHV 6 viremia Bronchial alveolar lavage that revealed *Stenotrophomonas maltophilia* and HHV6. Treatment was initiated with Foscarnet alone followed by combination ganciclovir. Despite aggressive intervention, he continued to be ventilator dependent with worsening pulmonary hypertension due to chronic lung disease. Eventually, the decision to cease supportive care was made in conjunction with the family 40 days after hospitalization.

This is the first report, to the best of our knowledge, of an atypical Omenn syndrome due to ADA deficiency with expansion of CD56^br^CD16− NK cells. Only 2 other patients with ADA-SCID and features of Omenn syndrome have been reported [9, 10]. The variability in the phenotypic spectrum of classic SCID-associated genes emphasizes the necessity of genotype-phenotype correlations.

## References

[1] Omenin G. S. (1965). Familial reticuloendotheliosis with eosinophilia. *The New England journal of medicine*.

[2] Kato M., Kimura H., Seki M. (2006). Omenn syndrome—review of several phenotypes of Omenn syndrome and RAG1/RAG2 mutations in Japan. *Allergology international*.

[3] Villa A., Notarangelo L. D., Roifman C. M. (2008). Omenn syndrome: inflammation in leaky severe combined immunodeficiency. *Journal of Allergy and Clinical Immunology*.

[4] Sottini A., Quiros-Roldan E., Notarangelo L. D., Malagoli A., Primi D., Imberti L. (1995). Engrafted maternal T cells in a severe combined immunodeficiency patient express T-cell receptor variable beta segments characterized by a restricted V-D-J junctional diversity. *Blood*.

[5] Sieverkropp A. J., Andrews R. G. A., Gaur L., Shields L. E. (2005). Chimerism analysis by sex determining region Y (SRY) and major histocompatibility complex markers in non-human primates using quantitative real-time polymerase chain reaction. *Tissue Antigens*.

[6] Hershfield M. S., Chaffee S., Sorensen R. U., Hirschhorn R., Gelfand E. W. (1993). Enzyme replacement therapy with polyethylene glycol-adenosine deaminase in adenosine deaminase deficiency: overview and case reports of three patients, including two now receiving gene therapy. *Pediatric Research*.

[7] Shibata F., Toma T., Wada T. (2007). Skin infiltration of CD56bright CD16- natural killer cells in a case of X-SCID with Omenn syndrome-like manifestations. *European Journal of Haematology*.

[8] Sanchez J. J., Monaghan G., Børsting C., Norbury G., Morling N., Gaspar H. B. (2007). Carrier frequency of a nonsense mutation in the Adenosine Deaminase (ADA) gene implies a high incidence of ADA-deficient Severe combined immunodeficiency (SCID) in Somalia and a single, common haplotype indicates common ancestry. *Annals of Human Genetics*.

[9] Dalal I., Tasher D., Somech R. (2011). Novel mutations in RAG1/2 and ADA genes in Israeli patients presenting with T-B- SCID or Omenn syndrome. *Clinical Immunology*.

[10] Roifman C. M., Zhang J., Atkinson A., Grunebaum E., Mandel K. (2008). Adenosine deaminase deficiency can present with features of Omenn syndrome. *Journal of Allergy and Clinical Immunology*.

